# Modified Tuber Starches as Sustainable Biopolymers for the Encapsulating Bioactive Compounds: A Comprehensive Review

**DOI:** 10.3390/polym17243257

**Published:** 2025-12-07

**Authors:** César Samaniego-Rafaele, Rebeca Salvador-Reyes, Grimaldo Quispe-Santivañez, Maritza Barriga-Sánchez

**Affiliations:** 1 Programa de Doctorado en Ingeniería Agroindustrial Mención Transformación Avanzada de Granos y Tubérculos Andinos, Universidad Nacional del Santa, Nuevo Chimbote, Ancash 02711, Peru; 2025818007@uns.edu.pe; 2 Facultad de Ingeniería, Universidad Tecnológica del Perú, Lima 150101, Peru; 3 Escuela Profesional de Ingeniería Agroindustrial, Facultad de Ingeniería, Universidad Altoandina de Tarma, Acobamba 120701, Peru; 4 Laboratorio de Compuestos Bioactivos, CITE Pesquero, Acuicola y Agroindustrial Callao, Instituto Tecnológico de la Producción, Callao 07001, Peru; mbarriga@itp.gob.pe

**Keywords:** biopolymers, sustainability, rheological properties, enzymes, fermentation, biocompatibility, dual modification, *Solanum tuberosum*, *Oxalis tuberosa*, *Ullucus tuberosus*

## Abstract

Modified tuber starches have gained relevance as innovative and versatile materials for the encapsulation of bioactive compounds, distinguishing themselves from synthetic polymers due to their biocompatibility, biodegradability, and tunable functionality. This review analyzes the effects of physical, chemical, and biochemical modifications on the composition and morphological, rheological, thermal, and techno-functional properties of tuber starches, as well as their development prospects as coating materials in encapsulation techniques such as spray drying, freeze-drying, electrospinning, and emulsification. The evidence reviewed indicates that modified tuber starches exhibit reduced retrogradation, higher thermal resistance, improved solubility, and better digestibility, facilitating their application as protective agents. The main challenges for their industrial implementation are identified and analyzed, including the standardization of processes, scalability, and the ambiguous regulatory framework. In the future, research in this area should be directed toward the optimization of “clean-label” methodologies and the valorization of non-conventional tuber sources, thereby consolidating the development of safer, more effective, and more sustainable encapsulation systems for the food industry.

## 1. Introduction

Starches are abundant biopolymers in nature [1], specifically within the plant kingdom, where they represent the second largest source of carbohydrates [2]. They are characterized by their role as an energy reserve in plants, their importance as a source of dietary carbohydrates for humans [3], and their ability to provide texture, viscosity, and consistency to various industrial and food products [4]. Starches are typically extracted from the endosperm or cellular vacuoles of sources such as potatoes, corn, wheat, and rice [5]. However, a current trend involves their extraction from non-conventional sources such as seeds, legumes, and fruits [6]. The botanical source of the starch is highly relevant as it affects the organization and size of the granules [7], the presence of tertiary compounds such as lipids, proteins, and minerals [8], and its techno-functional properties [9]. For instance, tuber starches possess flattened and ellipsoidal granules [10] that produce clearer gels with high swelling power [11], making them ideal for use as gelling and thickening agents [2]. In contrast, starches from cereals, such as amylaceous corn (*Zea mays*) and barley (*Hordeum vulgare*), exhibit spherical and oval shapes [12], which yield gels with high elasticity and viscosity [13], making them more suitable for application in products such as soups, pastas, porridges, stews, and other foods.

Structurally, starches comprise amylose and amylopectin chains, with concentrations typically ranging from 20–30% and 70–80%, respectively [14]. Experimental evidence confirms that high amylose content impacts texture and stability [15], while high amylopectin content alters viscosity and resistance to gel formation [16]. Starches are recognized for their applicability in various industries, including textiles, pharmaceuticals [17], packaging [18], and especially the food industry [19]. In the food sector, they are valued for their versatility as thickening, gelling, and stabilizing agents [20] due to their rheological properties, gelatinization capacity [21], and swelling and solubility characteristics [22]. However, in their native (raw) state, starches have technological and techno-functional limitations that prevent them from fully meeting industrial demands [23]. The main challenges they face include low thermal and shear resistance [24], a high tendency toward retrogradation [25], and sensitivity to pH changes [26]. Therefore, it is necessary to modify their morphological and structural properties through techniques and methods [27] that enhance their techno-functional characteristics [28].

In recent years, modified starches have gained increased commercial value [29], becoming the most widely used active ingredient in the food and health industries [24]. Their applications span various technological and engineering processes, including the microencapsulation of bioactive compounds [30], the development of edible films [15], and fat replacement in food matrices [31]. The primary modification techniques are physical [32] and chemical [33], although biochemical and biological modifications are now being employed to reduce energy consumption and environmental impact [34].

Modified starches hold significant potential in the modern food industry, particularly for their capacity as encapsulating agents for flavors, vitamins, polyphenols, and probiotics [35]. These modified biopolymers act as coating materials [36] to protect the desired compounds from degradation, control their release during digestion, and improve their shelf life [37]. They offer advantages over synthetic polymers due to their low toxicity, rapid biodegradability, cost effectiveness, and compatibility with other materials [38]. The modified starches commonly used for this purpose are derived from commercial cereals, tubers, and roots, with notable examples including potato (*Solanum tuberosum*) [39], corn (*Zea mays*) [40], cassava (*Manihot esculenta*) [41], and rice (*Oryza sativa*) [42]. However, from a technological standpoint, modified tuber starches exhibit superior characteristics for encapsulation due to the shape and structure of their granules [43], thermal properties [42], high paste viscosity [44], and process efficiency, which can reach up to 89.83% [45].

Recently, this approach has taken a new direction by utilizing starches from non-conventional tubers, such as oca (*Oxalis tuberosa*), mashua (*Tropaeolum tuberosum*) [46], achira (*Canna indica*) [47], and certain potato varieties [44,45] from the Andean region. The goal is to add value to crops from family farming and ensure food security [48]. These starches have become a reliable, and sometimes superior, alternative to their commercial counterparts from a technological perspective. This advantage is supported by their notable properties, such as their high amylose content, which can reach up to 35.6% [49], which is higher than that of potato (*Solanum tuberosum*) and cassava (*Manihot esculenta*), which typically ranges from 20% to 25% [50,51]. The B- and C-type crystallinity patterns [52] facilitate their dispersion in water and produce more transparent gels, unlike the A-type dispersion found in cassava (Manihot esculenta) and sweet potato (*Ipomoea batatas*) [53]. These starches exhibit a greater diversity of granule shapes, including ellipsoidal, oval, conical, and prismatic forms [54]. This combination of physical, chemical, and structural characteristics enables a range of innovative applications, such as bioactive compound encapsulation. Although still limited, these applications have demonstrated high efficiency, stability, and availability [55], underscoring the importance of applying appropriate starch modification methods and optimizing microencapsulation techniques to enhance their use.

In this context, this review aims to present the characteristics and properties of modified starches from both conventional and non-conventional tubers, focusing on their techno-functional properties, modification techniques, and development prospects as coating materials for the encapsulation of food compounds. The results obtained are expected to provide a better framework for future research and directly contribute to addressing technological challenges such as bioavailability, thermal instability, and protection of ingredients and/or bioactive compounds encountered with some conventional grain and root starches. This work also aims to foster the parallel development and advancement of science, ensure food security, and contribute to the Sustainable Development Goals (SDGs) of the United Nations.

**Figure 1:**
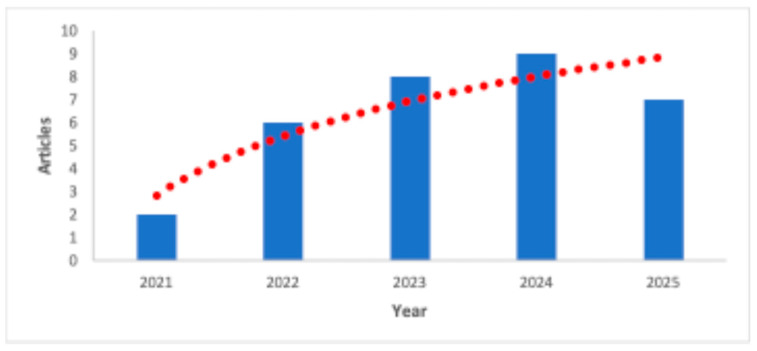
Annual scientific output regarding tuber starches and their application in the encapsulation of bioactive compounds.

A search was conducted in the *Scopus*, *ScienceDirect*, and *Google Scholar* databases using keywords such as: “*starch*” and “*modified*” and “*tubers*” and “*bioactive compounds*” and “*techniques*” and “*encapsulation*” and “*materials*” and “*technofunctional*”. Research published in the last five years (2021 to 2025) was prioritized due to an observed increase in the number of studies on these topics (Figure 1). This process yielded an average of 40 articles explaining the phenomena induced by modification on the structure and techno-functional properties of native starch, as well as the effects of the encapsulation technique type on the encapsulated compounds’ efficiency, protection, stability, and controlled release. This information was used to create tables to summarize and facilitate the interpretation of the results.

This article adopts a coherent and logical structure. The second section describes the composition, morphology, and functional properties of tuber-derived starches, as well as the physical, chemical, and biochemical modification techniques and effects. Subsequently, the third section analyzes recent advances in their application as encapsulating agents for bioactive compounds using contemporary techniques such as spray drying, freeze-drying, electrospinning, and emulsification. Finally, the fourth and fifth sections describe and examine the technological, regulatory, and nutritional challenges that guide the development of scalable and “clean-label” methods, the valorization of non-conventional sources, and integration with emerging technologies.

## 2. Tuber Starches: Characteristics and Modifications

### 2.1. Composition and Morphology

Tubers are a significant source of carbohydrates, primarily starch, which can constitute between 20% and 95% of their dry weight. This botanical group includes widely cultivated species such as taro (*Colocasia esculenta*) [56], potato (*Solanum tuberosum*) [57], arracacha (*Arracacia xanthorrhiza*) [58], yam (*Dioscorea* spp.) [59], and sweet potato (*Ipomoea batatas*) [60], as well as lesser-known sources such as ulluco (*Ullucus tuberosus*) and mashua (*Tropaeolum tuberosum*) [61].

**Figure 2:**
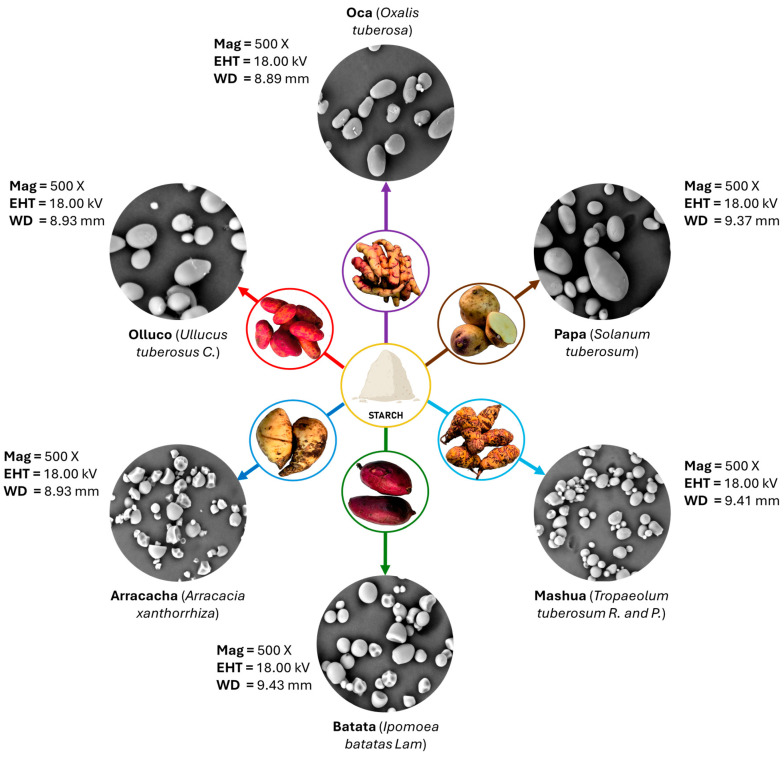
Morphology of the starch tuber granules.

Starches derived from these plant species are characterized by a high percentage of amylopectin and, in some cases, the presence of dietary minerals such as phosphorus and potassium [62]. From a morphological perspective, these starches exhibit wide structural diversity, with shapes ranging from round and elongated granules [63] to ellipsoidal and oval configurations [54], as shown in Figure 2. Their size can vary from 5.36 µm, as in mashua (*Tropaeolum tuberosum*) [61], to 61.8 µm, observed in certain potato (*Solanum tuberosum*) varieties [64].

These tuber starches display varying water and oil absorption capacities, with values typically ranging from 0.884 to 0.951 and 0.962 to 1.152, respectively [65]. Furthermore, these starches exhibit a gelatinization onset temperature of 58.3 °C [61], while viscosity can reach values of up to 11,600 mPa·s [60] (Table 1). These parameters directly influence properties such as digestibility, gelling capacity, and retrogradation [66].

All these characteristics confer notable versatility upon tuber starches, favoring their application in various technological areas [67], including nanoparticle synthesis [68], microencapsulate development [69], biofilm fabrication [70], and edible coating production [71]. 

**Table 1:** Composition and main functional properties of tuber starches.

Plant Material	Starch Content (%)	Amylose Content (%)	Amylopectin Content (%)	Gelatinization Temperature (°C)	Viscosity (mPa·s)	Reference
Taro (*Colocasia esculenta*)	93.55	17.89	75.66	74.52	5953.5	[56]
*Papa* (*Solanum tuberosum*)	85.50	29.90	20.00	58.50	NR	[72]
Arracacha (*Arracacia xanthorriza*)	98.40	34.31	NR	63.10	960.0	[58]
Yam (*Dioscorea bulbifera*)	77.30	16.60	83.40	94.10	7701.0	[59]
Olluco (*Ullucus tuberosus C.*)	54.48	23.92	NR	60.80	290.0	[61]
Batata (*Ipomoea batatas Lam*)	48.38	16.40	83.60	83.50	11,600.0	[60]
Mashua (*Tropaeolum tuberosum R. and P.*)	22.54	26.54	NR	61.02	370	[61]
Oca (*Oxalis tuberosa Mol.*)	32.78	24.38	NR	58.30	340	[61]

NR = No report.

### 2.2. Starch Modification Techniques and Their Effect on Tuber Starch Characteristics

Despite their great versatility and potential as biological materials in industry, starches in their native state often present application limitations [73]. Consequently, one or more of their physical and chemical properties must be modified [30] to achieve desired characteristics and enhance their technological value for the food and pharmaceutical industries [28]. The key properties targeted for modification include viscosity [74], solubility, and thermal stability [75].

**Figure 3:**
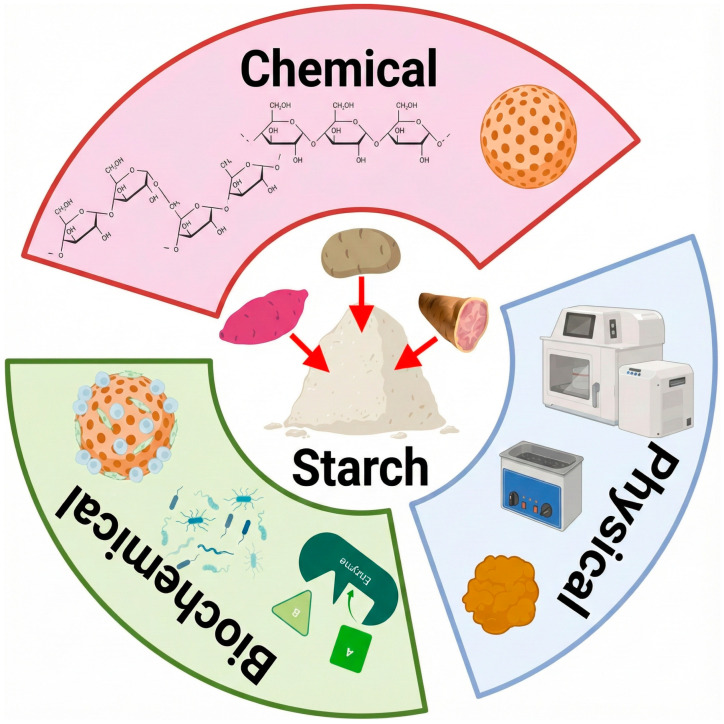
Key techniques for the modification of tuber starch.

As illustrated in Figure 3, starch modification is generally performed using physical, chemical, and biochemical methods. Physical methods directly affect starch viscosity, solubility, and granule size [76]. Chemical methods, on the other hand, operate at a molecular level [77], modifying the thermal and rheological properties of the biopolymer. Biochemical methods focus on the use of enzymes and microorganisms to modify the structure of amylopectin chains and the amylose content [78], resulting in improved digestibility and altered granular morphology. Currently, combined strategies, either dual or triple, and homogeneous or heterogeneous in nature, are being employed to confer specific structural and functional properties [79] for targeted technological processes or applications.

The operational details of each methodological classification are detailed below to elaborate on the scope of amylaceous modifications, exploring changes in morphological, thermodynamic, and nutritional properties.

#### 2.2.1. Physical Modifications

**Figure 4:**
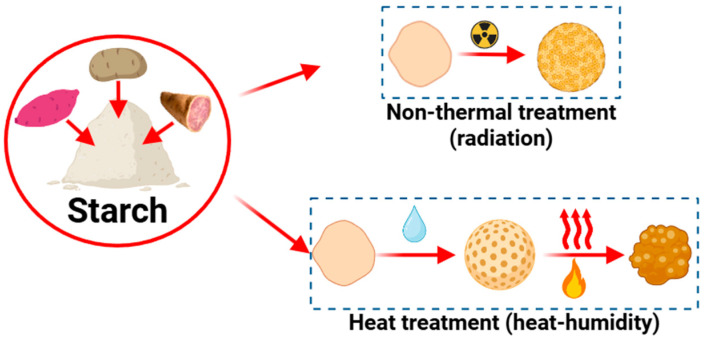
Physical modification methods (thermal and non-thermal) applied to tuber starches.

This involves altering the starch structure through thermal and non-thermal treatments (Figure 4). Thermal methods include pregelatinization, heat-moisture treatment, annealing, and microwave heating [80], whereas nonthermal methods include milling, sonication, pulsed electric fields, and high-pressure treatments [81]. These procedures are characterized as relatively simple, economical, rapid, and environmentally friendly [82]. It is important to mention that modified tuber starches fall within the “clean label starch” group, as they pose no health risks to consumers and are entirely produced with non-contaminating, natural ingredients [83].

Non-thermal methods tend to produce cracks, pores, and channels in the starch granules [84], whereas thermal methods cause molecular aggregation, forming homogeneous structures with low dispersion [85]. These changes in the granules and their organization influence the crystalline structure [19], gelatinization thermal properties [86], rheological properties, such as viscosity and retrogradation [87], techno-functional properties, such as solubility, swelling power, hydration, and adhesiveness [74], and mechanical properties. Comparing these characteristics with conventionally modified grain and cereal starches, tubers show greater paste clarity, moisture retention, and viscosity changes through treatments such as HMT and microwaves. Cereal starches tend to undergo changes related to granule integrity and molecular compaction, affecting their gelatinization and retrogradation properties, which are key aspects for their future technological applications.

The degree of modification achieved by this method depends on variables such as temperature (T°), pressure (P), time (t), moisture, and the initial concentration of the native starch [88]. Table 2 presents some examples of the application and effects of these modifications on tuber starches.

As shown in Table 2, physical treatment induces significant modifications in the thermal properties of tuber starches. Among the most relevant changes are increased heat resistance [45], gelatinization temperature [77], and a decrease in free amylose content [45,77]. Furthermore, alterations have been observed in fundamental rheological properties, such as viscosity [77,89], and in physicochemical properties, such as retrogradation [45,89]. By changing gelatinization and thermal properties through the structural disorganization of the granules, this modification promotes the formation of more stable matrices for encapsulation, with the capacity to protect and control the release of active ingredients, improving their efficiency through microencapsulation techniques such as spray drying [42]. This promotes gel formation and improves paste clarity, leading to the formation of denser and more stable matrices, ideal for the encapsulation of bioactive compounds [84].

Currently, these modified tuber starches can also be obtained through homogeneous dual methods, in combination with extrusion and annealing [90], as well as heterogeneous methods that include chemical processes such as acetylation, acid hydrolysis, and oxidation [91]. These transformations and characteristics open new possibilities for applications in the food and non-food sectors.

#### 2.2.2. Chemical Modifications

This widely developed method involves altering the molecular structure of native starches through chemical reactions that affect their techno-functional and structural properties [92]. Oxidation is one of the main types of modification, which involves introducing carbonyl (–C=O) and carboxyl (–COOH) groups into its structure, improving its thickening and stabilizing properties [93]. Another relevant technique is esterification, which consists of incorporating ester groups (–COOR) into the glucose chains using agents such as octenyl succinic anhydride (OSA), resulting in starches with better hydrophobicity and emulsifying capacity [76]. Finally, phosphorylation and cross-linking are used to introduce phosphate groups using agents such as sodium trimetaphosphate (STMP) and sodium tripolyphosphate (STPP), significantly improving parameters such as thermal stability, viscosity, and solubility [94]. The effectiveness of this modification method is influenced by factors such as the type of chemical reaction, the functional groups added, the degree of substitution, and the reaction conditions [93].

This type of modification alters the molecular weight distribution and branching patterns of starch molecules, resulting in their degradation [95]. Another perceptible change occurs on the granule surface, with an increase in roughness and the formation of pores and cracks [96].

**Figure 5:**
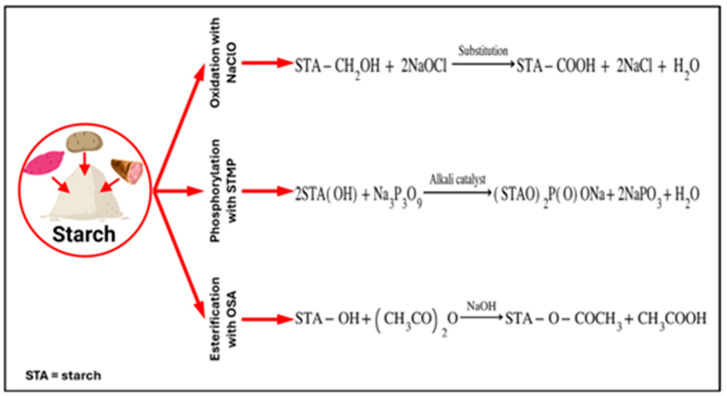
Chemical modification methods for the preparation of tuber starches.

Figure 5 presents the main chemical modification methods used to prepare tuber starches.

Despite their multiple advantages, the acceptability of these starches in the food sector has begun to decline due to growing concerns about their potential side effects on health [97], the generation of residual by-products during their production [98], and the poor development of legislation establishing clear criteria for their application in food matrices [28]. In this context, optimizing modification methods, evaluating their implications for human physiology, and analyzing their environmental impact are essential to ensure their safety and industrial viability [99].

Table 3 presents some examples of the application and effects of these modifications on tuber starches.

As shown in Table 3, the modification of starches from traditional tubers, such as potato (*Solanum tuberosum*) and taro (*Colocasia esculenta*), results in increased thermal stability [100,102] and peak viscosity [94,103]. This favors the formation of denser and more cohesive matrices, facilitating the retention of volatile compounds and making them ideal for thermal encapsulation processes, such as spray drying [84]. In contrast, starches derived from Andean tubers, such as oca (Oxalis tuberosa) and ulluco (Ullucus tuberosus) [104], which were modified with OSA, experience a decrease in thermodynamic parameters, such as enthalpy, pasting temperature, and gelatinization temperature. This reflects lower energy and structural stability, along with greater emulsifying capacity and high oil retention, making them effective for encapsulation [104]. These characteristics may facilitate the release of compounds into the medium by generating matrices with weaker structures, thus recommending their incorporation into low-temperature freeze-drying encapsulation processes.

Other chemical modification alternatives include homogeneous dual methods, such as esterification with OSA and cross-linking with STMP [94], which have a significant effect on properties such as viscosity and stability, conferring high potential as binding, thickening, and emulsifying agents in the food industry [105]. Another alternative is modification by heterogeneous methods, such as those integrating dry heat treatments and hydrothermal processes, which provide better thermal stability and reduce the need for surfactant agents [76]. Despite the numerous benefits and process versatility offered by these methods, factors such as genetic variability, treatment order, and reaction type still significantly affect the final physical and techno-functional properties of the modified starches [99].

#### 2.2.3. Biochemical Modifications

**Figure 6:**
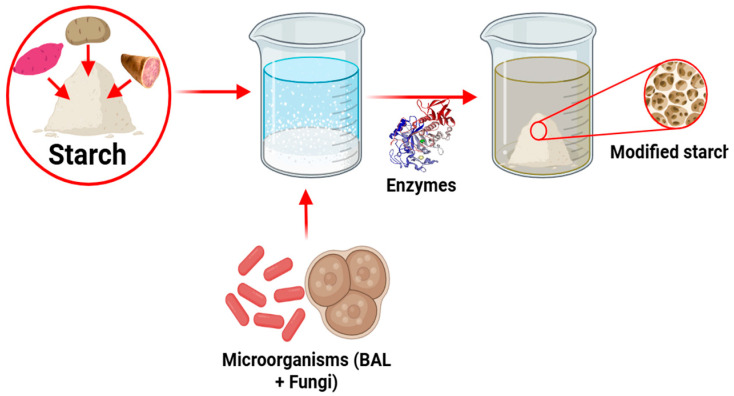
Biochemical modification of tuber starches.

This is a biotechnological method that uses enzymes and microorganisms (Figure 6) to alter the structures and physicochemical properties of native starches to diversify their applications in industry and food [76]. Compared with chemical starch modification methods, this method is considered one of the most economical and ecological alternatives and is positively valued for its functionality and potential for developing innovative products [43].

The main enzymes used in this method are α-amylase, β-amylase, glucoamylase, isoamylase, pullulanase, and amylosucrase [106]. These enzymes tend to alter the molecular mass distribution and branched chain length of starches, which affects the amylose/amylopectin ratio [107] and the degree of crystallinity [108]. These modifications result in starches with improved digestibility, higher resistant starch content, and enhanced prebiotic effects, favoring their functionality in both industry and health [107]. However, regarding the use of microorganisms in this modification method, the use of fermentative bacteria and fungi is notable [109]. Lactic acid bacteria, such as *Lactobacillus calbbiosus*, *Streptococcus lactis*, and *Corynebacterium* sp. [110], are the main bacteria, while fungi include *Saccharomyces cerevisiae* and tape yeast [111]. These microorganisms act on the surface and granular structure of the starches, generating pores through erosion and rupture that facilitate the entry of enzymes and their degradation [112]. Another important change occurs in the crystalline structure of the starches, which can increase because of amorphous region degradation and short chain alignment into ordered structures [113]. Additionally, these modifications significantly affect the thermal properties, viscosity, gelatinization [114], swelling power, and solubility.

Table 4 presents the parameters and effects of biochemical modifications on the characteristics of tuber starches.

As observed in Table 4, many of the changes experienced in the properties of tuber starches are related to decreased values of viscosity [118,119], enthalpy [115,116], amylose content, and molecular weight [116,117], which can be attributed to enzymatic and microbial interactions with their molecular structure. The reduction in viscosity and enthalpy values was also experienced when applying enzymes such as α-amylase and glucoamylase [120], as they break glycosidic bonds, generating smaller fragments and modifying the structural order of the starch, resulting in changes in rheological and thermal properties. The reduction in amylose content and molecular weight [121] is attributed to the enzymatic hydrolysis of polymeric chains, which directly affects functional properties such as retrogradation, texture, and digestibility. On the other hand, characteristics such as hardness experienced an increase, most likely due to the recrystallization of short amylose and amylopectin chains [122] or interaction with other compounds (lipids or proteins) that generate complexes with greater structural cohesion. The changes brought about by this modification method generate starches with greater resistance to digestion, facilitate the formation of fine microparticles [19], and create stable and manageable matrices, thereby expanding their technological applications to techniques such as encapsulation by spray drying [123].

However, despite their great potential, the implementation of this biochemical modification method faces technical constraints such as the limited diversity of structures that can be generated with a single enzyme, or the costs implicated in their application and scalability [124]. To address these challenges, current research seeks to overcome these restrictions by adopting dual methods that combine homogeneous approaches such as mixed enzymes combining lactic acid bacteria and yeasts such as *Pichia membranifaciens* [116] or heterogeneous approaches such as physical-enzymatic techniques [118]. These innovations aim to overcome current barriers, ensuring greater efficacy in the controlled modification of starches for industrial applications.

## 3. Encapsulation with Tuber Starches

Encapsulation is an advanced engineering technique at the macro, micro, and nano scale, designed to protect sensitive food ingredients, such as antioxidants, probiotics, vitamins, and other bioactive compounds [125]. The most commonly used wall materials in this technique are polysaccharides, lipids, and, in certain situations, proteins, which have demonstrated notable efficacy in protecting active principles against environmental factors like light, oxygen, humidity, and heat [124]. This technique allows for the control of critical parameters, such as stability, bioavailability, and release kinetics, within food matrices using specifically selected wall or coating materials [126]. Recent studies have shown that the synergistic interaction between wall materials and active principles can optimize the performance of encapsulation systems and broaden their industry applicability [38]. However, it is important to note that there is no standardized encapsulation procedure, as each technique and material presents advantages and limitations, with the physical and chemical properties being what determines the choice of the most suitable process [127].

**Figure 7:**
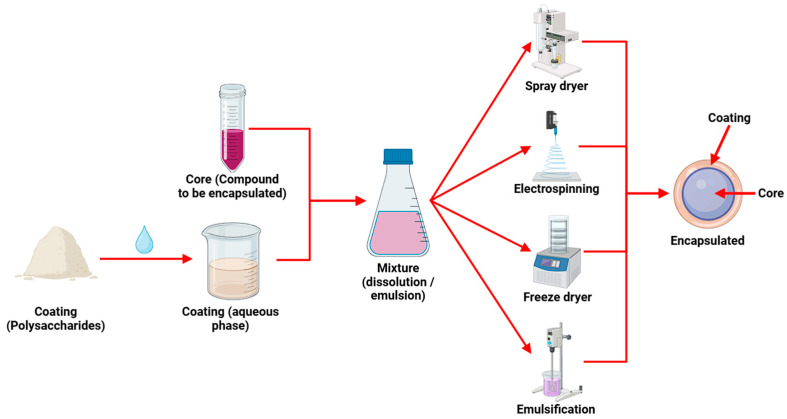
Encapsulation techniques using tuber starches.

With respect to modified starches derived from tubers, these are frequently used in the encapsulation of various compounds, including probiotic agents (lactic acid bacteria) [45], macromolecules, chemical compounds (organic acids and proteins) [55,102], bioactive compounds (phenolic and sulfur compounds) [46,101,128], and aromatic compounds (essential oils) [100]. This biopolymer has demonstrated notable protective capacity, yielding results comparable to those obtained with thermostable synthetic polymeric materials, such as epoxy resin or polycaprolactone (PCL) [129]. Due to these properties, modified starches are positioned as a promising material for the development of functional coatings, applied using advanced techniques such as spray drying, freeze-drying, electrospinning, and nanoemulsification [37] (Figure 7).

The formation of the aqueous coating phase and its subsequent incorporation with the active agent is an essential step in using starches to obtain encapsulates, a process resulting in the formation of solutions and emulsions. Although the starch concentrations required for the aqueous phase can vary significantly according to their biological origin, the use of optimal amounts is suggested to ensure the formation of solid microspheres [130] and maintain adequate viscosity [131]. The efficacy and efficiency of these biopolymers are ultimately determined by variables such as the degree of starch modification, the encapsulant-to-active agent ratio, and the operational conditions [46].

From a technological perspective, these systems are recognized for their ability to transport, protect, and controllably administer bioactive components such as fatty acids, phenolic compounds, carotenoids, flavors, essential oils, vitamins, bactericides, and enzymes [37]. These characteristics are attributable to the techno-functional properties of starch, such as swelling power, thermoreversible behavior (gelatinization), and affinity for aqueous and lipid phases that these starches exhibit [65]. Scientific studies on simulated in vitro digestion conditions demonstrated the ability of these systems to modify the insulin response [132] and reduce the glycemic index and glucose absorption [133], which opens new prospects for the formulation of healthier foods and demonstrates the technological potential of these biopolymers.

### 3.1. Spray Drying

**Figure 8:**
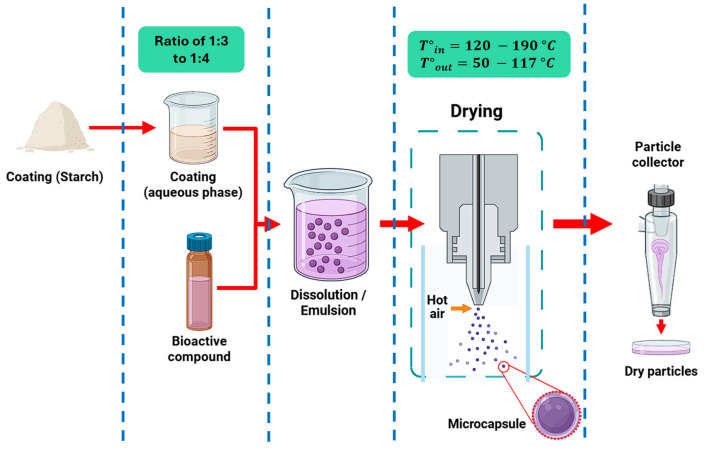
Microencapsulation by spray drying using tuber starches as coating materials.

Microencapsulation is one of the most widely used microencapsulation techniques in the food and pharmaceutical industries, noted for its cost-effectiveness, simplicity, versatility, and high efficiency [134]. This process involves the transformation of liquid matrices, such as emulsions, solutions, or colloidal suspensions, into micrometric solid particles with polymorphic morphology through various drying technologies [135] (Figure 8). Among its main operational advantages is its ability to operate in a continuous regime, allowing for the production of large volumes of encapsulates at minimal costs [136].

The primary purpose of this technology is to protect thermolabile or sensitive compounds from adverse environmental factors such as thermal fluctuations, humidity changes, and oxidation phenomena [137]. Another characteristic of this technique is its capacity to optimize the functionality of encapsulated compounds through the controlled release of the active principle, manipulation of physicochemical properties, and increased stability during storage [138]. An additional benefit lies in its ability to mask the undesirable sensory characteristics of nutraceutical compounds, thereby improving their consumer acceptability [139].

Currently, spray drying microencapsulation has become a key tool in the food sector to utilize bioactive compounds such as carotenoids, fatty acids, and phenolic compounds, which have been shown to possess relevant functional properties, including antioxidant, antimicrobial, anti-inflammatory, and anticancer effects [140]. Because each metabolite presents unique physicochemical characteristics for its encapsulation, the food sector must consider critical variables, such as the origin and purity of the bioactives, the molecular weight and chemical affinity of the encapsulant [141], the hydrophobicity/permeability of the wall materials [142], the concentration and compatibility of the system components [143], and the equipment’s operational parameters, to collectively optimize the encapsulation efficiency, stability, and final bioavailability of the active compound.

Table 5 presents some examples of the application of the encapsulation technique using tuber starches via spray drying.

As observed in Table 5, the application of starches extracted from tubers through physical and chemical modifications provides high encapsulation efficiency [102,144] and the formation of structures with spherical morphology [45,55], with diameters ranging from 10 to 60 µm [100]. Similarly, parameters such as water activity [101], hygroscopicity [46], and moisture [102] have been reported to have reduced values.

This behavior can be attributed to the introduction of functional groups through processes such as esterification [46,100,101], which allows for greater affinity toward both polar and non-polar compounds, positively influencing encapsulation efficiency. The size and spherical morphology of the microencapsulates appear to be closely related to the botanical origin of the starch granules used [145] and the physical processes they underwent. Finally, their low hygrometric properties reflect not only the quality of the process but also the system’s ability to prolong shelf life and conserve the stability of the encapsulated compounds [101].

### 3.2. Freeze-Drying

Freeze-drying is considered one of the most widely used technologies in encapsulation techniques because of its high potential for preserving thermolabile compounds such as biomolecules, cells, and other sensitive materials [146]. In the food field, its application has demonstrated particular efficiency in protecting compounds unstable to heat and oxygen, such as pigments, proteins, and microorganisms [147], through the application of polymeric matrices based on lipids, proteins, and starches as encapsulants [148]. The quintessential coating material is maltodextrin due to its good water solubility and low moisture absorption; however, its low emulsifying capacity [149] demands the application of new coating materials with greater biocompatibility with the cores and the ability to protect the physical and sensory characteristics of the component [150].

**Figure 9:**
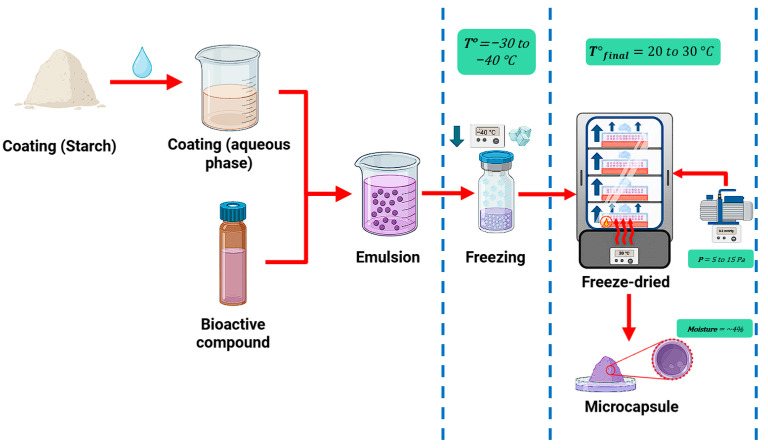
Microencapsulation by freeze-drying using tuber starches as wall materials.

The freeze-drying encapsulation process (Figure 9) involves three primary stages: rapid freezing of the matrices at cryogenic temperatures, primary drying or sublimation under vacuum, and secondary drying or desorption to remove unfrozen or bound water [151]. It is important to consider solute concentration, core-to-matrix ratio or matrix composition, and microstructure as process parameters [152].

Despite the relative simplicity of its process, this method exerts high thermal and osmotic stress on the components of the matrix intended for encapsulation, which can generate structures with irregular and porous shapes [153]. However, freeze-drying presents limitations for industrial applications due to its low scalability, high energy consumption, prolonged process times, and limited production capacities [154]. Table 6 presents some examples of the application of modified starches in microencapsulation processes by freeze-drying.

### 3.3. Electrospinning

**Figure 10:**
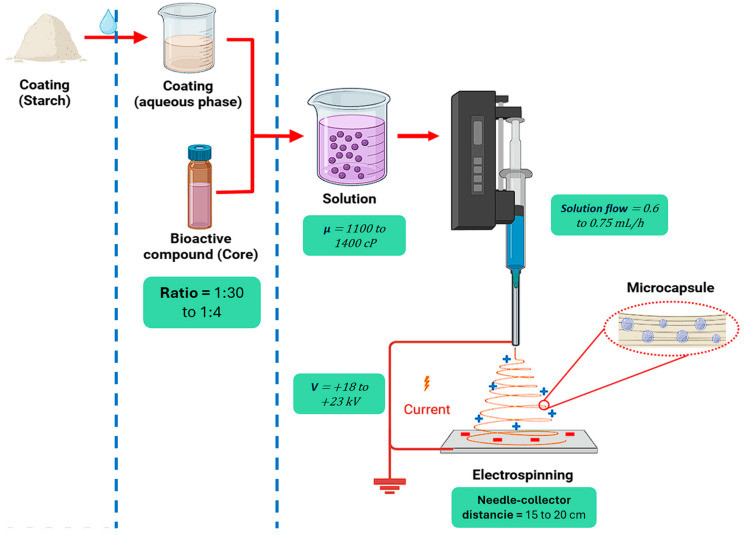
Microencapsulation by electrospinning using tuber starches as wall materials.

Also referred to as “wet spinning,” electrospinning is an emerging technique used to protect and release bioactive compounds through the production of polymeric microstructures, with applications in food science, tissue engineering, and biomedicine [155]. This method is characterized by the formation of continuous fibers with nanometric diameters using electrostatic forces that stretch natural or synthetic polymers [156]. Its efficiency depends on intrinsic factors, such as viscosity, conductivity, and solution concentration [157], and extrinsic factors, such as voltage, needle/collector distance, feed flow rate, and humidity and temperature [158]. As shown in Figure 10, the process technically begins with the formation of a viscoelastic solution that is controllably injected into a vial connected to a high-voltage generator via a peristaltic pump, inducing the formation of a fibrous “jet” that is deposited on a collector for subsequent drying [159].

Among the main natural polymers used in this technique, native and modified starch possess a significant technological advantage due to their amylose chain content and linear structure, which facilitates the formation of continuous and consistent fibers [156]. Other characteristics to consider are its biocompatibility, biodegradability, nontoxicity, and low cost of preservation, making it ideal compared to synthetic and animal-based alternatives [160].

Encapsulates obtained by this method exhibit notable resistance to adverse environmental factors, such as temperature, light, and oxidation, in addition to high efficiency in maintaining the active principle’s stability, bioavailability, and bioactivity [159]. However, its industrial-scale implementation is restricted by technical problems such as needle clogging, limited material selection, low industrial scalability, and low productivity [161]. Table 6 presents some examples of the application of modified starches in microencapsulation processes by electrospinning.

### 3.4. Emulsification

**Figure 11:**
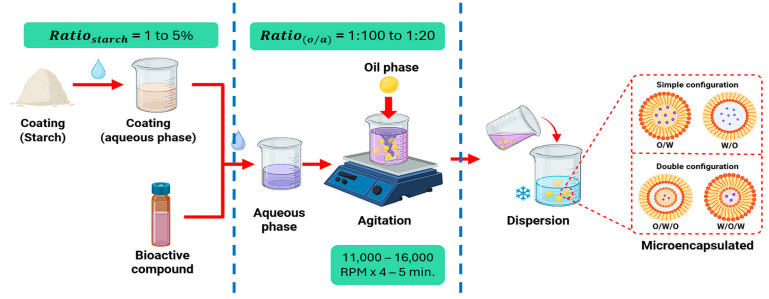
Microencapsulation by emulsification using tuber starches.

Microencapsulation by emulsification with starches is a key emerging technique in the food industry that is designed to improve the stability, bioavailability, and controlled release of bioactive compounds [162]. As shown in Figure 11, this physicochemical process is based on the formation of colloidal systems through the interaction of two or more immiscible fluids (continuous and dispersed phases) using surfactants and homogenization technologies [163]. Commercially, water and oil are the most used fluids in emulsification processes. They can be present in simple configurations, such as O/W (oil/water) and W/O (water/oil) [164], or in their double form, such as O/W/O (oil/water/oil) and W/O/W (water/oil/water) [165]. Starches serve as emulsifiers, with those from rice (*Oryza sativa* L.) and corn (*Zea mays* L.) being the most applied in this process [166]. Currently, the use of tuber starches in their modified form is proposed because of their hydrophobic characteristics, hydrophilic groups, and small grain morphology, which gives them high emulsifying capacity and thus makes them ideal for this type of process [167].

Among its main advantages, this technique stands out for its low preparation complexity and the ability to enhance functional properties such as digestibility and antimicrobial and antioxidant activity [168].

Although this method is considered thermodynamically stable, the obtained microencapsulated systems present chemical vulnerability to environmental factors (temperature, pH, etc.), producing phenomena such as flocculation and oxidation, which in turn compromise functionality [169]. However, high starch concentrations cause an increase in the viscosity of the aqueous phase, requiring the addition of high concentrations of surfactant to stabilize the emulsion [170].

Among the main technological limitations of this technique, the use of modified starches can cause low surface charge, reducing electrostatic repulsion between particles [145]. This can favor the premature release of bioactive compounds into the medium, as well as high particle size and morphology heterogeneity [171]. These characteristics could restrict their application in certain food matrices and open new research perspectives aimed at improving their processes.

Table 6 presents some examples of the application of modified starches in microencapsulation processes by emulsification.

**Table 6:** Application of freeze-drying, electrospinning, and emulsification encapsulation techniques using tuber starches.

Tubers	Encapsulation Technique	Parameters	Properties of the Microencapsulates	Ref.
Potato (*Solanum tuberosum*)	Freeze-drying	- WM: Acid-hydrolyzed starch - CM: gallic acid (0.1, 1.0, 5.0, and 10.0 g GA/100 g starch) - Freezing: Fast - Sublimation: vacuum (final moisture: 4%) - Processing time: ~24 h.	↑ Encapsulation efficiency (70–84%) ↑ Pore volume (2.4 × 10^−3^–9.5 × 10^−3^ cm^3^/g) ↑ Operating cost (4 × higher than spray drying) ↓ Water activity (0.059–0.090) ↓ Surface area (0.632–1.225 $m^{2}$/g) Irregular morphology.	[55]
Tigernut (*Cyperus esculentus*)	Freeze-drying	- WM: Modified tigernut starch + inulin - CM: Tigernut milk - Pre-freezing: 40 °C × 24 h. - Freeze-drying time: 48 h (vacuum)	↑ Thermal stability (>346 °C) ↑ Vitamin C content (3.17 ± 0.05 mg/100 g) ↑ Carbohydrate content (65.1%) ↓ Acidification (pH 6.88–6.99) ↓ Particle size (~1.01 µm) ↓ Mesophilic/mold growth (<10^4^ UFC/mL) Smooth and spherical morphology	[172]
Sweet potato (Lpomoea *batatas*)	Electrospinning	- WM: Formic acid-modified starch. - CM: Red onion skin extract, 9% (0, 3, and 6 years) - Voltage: +18 kV - Needle-collector distance: 20 cm. - T°: 22 ± 2 °C - Flow rate: 0.75 mL/h - RH: 43 ± 5%	↑ Encapsulation efficiency (67–78%) ↑ Fiber diameter (251–611 nm) ↑ Antioxidant activity (92–96.9%) ↑ Lipid medium release (44–100%) ↑ Thermal resistance at 100 °C (51.6–95.4%) ↓ Hydrophilic release (<10%) Antimicrobial activity against *E. coli* and *S. aureus.*	[173]
Potato (*Solanum tuberosum*)	Electrohilado	- WM: Native potato starch (3%, 5%, 10%, 15%, and 20%) - CM: Curcumin (0; 0.5; 0.75 y 1%) - Voltage: +23 kV - Solvent: Formic acid - Needle-collector distance: 15–20 cm. - T°: 22 ± 2 °C - Flow rate: 0.60 mL/h - RH: 45 ± 5%	↑ Encapsulation efficiency (79.01–96.20%) ↑ Conductivity (1.2–4.8 mS/cm) ↑ Viscosity (1100–1400 cP) ↓ Thermal loss at 180 °C × 2 h. (17.40–34.08%) ↓ Fiber diameter (108–142 nm) ↓ Antioxidant activity: ABTS (11–45%) Homogeneous cylindrical morphology and formation of low-concentration bead	[128]
Oca (*Oxalis tuberosa*)	Emulsification	- Emulsion type: O/W - Dispersed phase: canola oil - Continuous phase: Water + OSA-modified oca starch (1%, 2.5%, and 5% *w*/*w*) - Homogenization: 16,000 rpm for 5 min	↑ Stability (40 days) ↑ Emulsification index (0.6–0.8) ↑ Emulsion droplet size (76.5–92.5) Homogeneous morphology with the formation of a dense layer around the oil droplets.	[174]
Potato (*Solanum tuberosum*)	Emulsification	- Type: O/W - Dispersed phase: paraffin oil. - Continuous phase: Distilled water (with 0.02% sodium azide) + OSA and HMT-modified starch - Homogenization: 11,000 rpm for 4 min.	↓ Viscosity (4.23–10.73 cP) ↑ Emulsification Index (0.41–1.00) ↑ Stability at 400 mg/mL ↑ Fat Binding Capacity (186.57–230.65%)	[76]
Sweet potato (*Ipomoea batatas*)			↓ Viscosity (3.57–15.23 cP) ↑ Emulsification Index (0.63–1.00) ↑ Stability at 400 mg/mL ↑ Fat Binding Capacity (219.52–261.07%)	

**Note**: WM = wall material; CM = core material.

## 4. Advantages and Challenges of Using Modified Tuber Starch in the Encapsulation of Bioactive Compounds

Modified starches derived from tubers, such as potato (*Solanum tuberosum*), cassava (*Manihot esculenta*), sweet potato (*Ipomoea batatas*), and yam (*Dioscorea* spp.), have emerged as advanced materials for the encapsulation of bioactive compounds, significantly overcoming many of the technological limitations of their native counterparts [175]. These modified starches are generally obtained through physical [76], chemical [77], biochemical [78], techniques, or combinations thereof, achieving significant effects on key characteristics such as protection capability against adverse conditions [159], increased bioavailability, and precision in controlled release during encapsulation [126]. However, their industrial implementation for obtaining encapsulates and their food applications still faces significant challenges related to the optimization of operational conditions, production scalability, and compliance with health and legal regulations [176].

### 4.1. Technological Advantages and Challenges

Among the significant advantages of using modified tuber starches in encapsulation processes is their ability to preserve the thermal [55,172,173,177] and oxidative [144] stability of sensitive compounds, such as phenols [46,101], proteins [102,177], and essential oils [100,144], significantly reducing their degradation during processing and storage. Similarly, an increase in the viability of encapsulated microorganisms has been observed [45,178]. Unlike commercial cereal starches, such as corn and wheat, or derivatives, such as maltodextrin, which are characterized by short amylopectin chains associated with lipids and proteins and type A crystalline patterns [179], modified tuber starches have a higher proportion of long amylopectin chains, minerals, such as phosphorus, and type B crystalline patterns [180]. This unique composition translates to lower rapid digestibility and a higher resistant starch content, which, combined with the wide range of granule shapes and sizes [66], lead to improved gelling properties, paste clarity, and swelling power [179]. As a result, homogeneous matrices with greater stability and low dispersion are formed [85]. These properties favor the formation of films and nanoparticle structures [181] and are crucial for the encapsulation and release of bioactive compounds. This natural origin confers biodegradability properties [182], positioning them as a sustainable alternative to other synthetic encapsulating materials and aligning with current eco-friendly production trends. Finally, the capacity of native and modified tuber starches to adapt to food products, such as meats [183], beverages, and dairy products [184], without compromising the physical, chemical, and sensory characteristics of the final product must be mentioned.

Regarding technological challenges, we can highlight the limited number of tuber alternatives applicable at an industrial level due to low efficiency and stability in operations [100,101,177], requiring advances in process engineering, material characterization, and adaptable formulation design. Another factor to consider is the replicability of industrial-scale modification and encapsulation processes, which are directly affected by mass production costs, process times, and the implementation of specialized equipment and machinery [99,161]. Likewise, biocompatibility with complex food matrices represents a constant challenge, as unwanted interactions can alter the final product, triggering sensory problems (e.g., texture, color, and flavor) [185] and affecting the release kinetics of the bioactive compound [168]. Another aspect to consider is the lack of standardized modification protocols, which are already developed for traditional products such as corn (*Zea mays*) and wheat (*Triticum* spp.) and are supported by American agencies such as the Food and Drug Administration (FDA) [186] and European Parliament and Council regulations [30]. Finally, despite the natural origin and biodegradable characteristics of these starches, poor practices in certain techniques and modification processes can generate toxic byproducts that are harmful to health and the environment [187], demanding a rigorous assessment of their consumption safety and environmental regulatory compliance before their industrial development.

### 4.2. Nutritional Advantages and Challenges

Among the main nutritional advantages, their influence on the bioavailability, stability, and functionality of essential nutrients stands out [126]. This effect is attributed to the alteration in physicochemical properties, such as solubility, hydrophobicity, and lipophilicity [76], induced by techniques such as cross-linking in starches. These alterations allow for a controlled release of active principles, delaying starch (encapsulant) digestion and facilitating better absorption of bioactive compounds (core) in the gastrointestinal tract [168]. These polymers generate a barrier against degrading factors such as oxygen, pH, and light [125] and are effective in protecting fragile compounds such as vitamins, polyphenols, and polyunsaturated fatty acids [141]. A notable example is the application of modified starches (RS4) in the encapsulation of essential oils, where they prevent their oxidation and preserve their cardiovascular benefits [188]. The prebiotic potential of certain resistant starches (RS3 or RS4) obtained through thermal [76] or chemical [101] modifications is another relevant characteristic. These starches can serve as substrates for the gut microbiota [189], generating prebiotic effects, such as insulin resistance and reduced inflammation [190], which can lead to a lower glycemic response in encapsulated foods, especially for the population with diabetes, hypertension, and obesity.

The main nutritional challenges faced by encapsulates with starches depend on the type of modification they undergo, the compound to be encapsulated, the application medium, and the intended purpose as a final product [191], making it essential to balance functionality, safety, and nutritional value [109]. Thus, the use of starches as a coating material can reduce the nutrient absorption rate because starch interferes with the accessibility of digestive enzymes to co-encapsulated proteins [192] and lipids [193], which requires precise adjustments of the food matrix to optimize controlled release. Another relevant challenge is overprocessing derived from aggressive starch modifications, such as those produced by high degrees of esterification [194] or high processing temperatures [195], which can generate indigestible encapsulates with irregular morphology and altered chemical structures of their components [196]. Finally, the effect of the interaction of some encapsulates with dietary minerals, such as calcium or zinc [197], must be considered because their binding generates non-absorbable complexes, which can limit their application in enriched formulations. Likewise, the natural content of minerals, such as phosphorus, in tubers, such as potato or sweet potato, must be evaluated, as some varieties have contents exceeding 700 ppm [198], which can negatively affect the quality and stability of foods derived from their starches.

## 5. Trends and Future Perspectives

**Figure 12:**
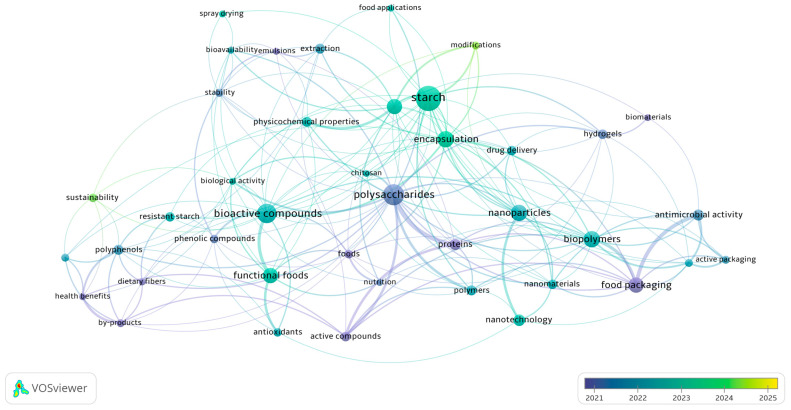
Keyword co-occurrence network (n ≥ 7) in publications on encapsulation of bioactive compounds with modified tuber starches over time (2021–2025). Note: Visualization was generated with VOSviewer v1.6.20 from ScienceDirect records (July 2025) using the query: TITLE-ABS-KEY (“starch” AND “modified” AND “tubers” AND “bioactive compounds” AND “techniques” AND “encapsulated”).

As shown in the publication impact network map (Figure 12), between 2021 and 2025, many applications of modified tuber starches were oriented toward the development of “food packaging,” “edible films,” “agriculture,” and the study of their composition based on their “proteins,” “dietary fiber,” or “phytochemical” content. However, between 2024 and 2025, the scientific trend took a new direction, focusing on evaluating the nutritional functionality and probiotic behavior of starches, denoted by the appearance of terms such as “functional foods,” “bioactive compounds,” “bioavailability,” and “biological activity.” On the other hand, from a technological viewpoint, the development of various techniques using starches as a protective barrier to preserve compounds sensitive to external factors can be observed, examples being the appearance of terms like “encapsulation,” “biopolymers,” “nanomaterials,” “delivery systems,” and “biocompatibility.” Finally, terms like “sustainability” are observed, denoting the growing concern of the academic area to maintain production and technological development focused on the proper management of resources and environmental care.

Although concepts such as “encapsulation,” “modified starches,” and “bioactive compounds” are not new in the scientific literature, their consolidation over the last decade has laid the groundwork for exploring new techniques based on non-conventional processes. This includes the application of emerging technologies such as cold plasma [199], ozone treatments, electron beam irradiation, and microfluidics [200]. Although these technologies were not specifically designed for treating tuber starches, they are safe and environmentally sustainable alternatives. However, it is crucial to note that currently, equipment availability, high energy consumption, and high operational costs represent a significant challenge for the transfer of these technologies to an industrial scale. However, it is important to note that these advances are also closely linked to the use of alternative sources, such as plant byproducts from the food, agricultural, and livestock industries. These byproducts gain prominence as sources of macronutrients, bioactive compounds, and biopolymers, positioning themselves as an innovative solution in food science.

Regarding the encapsulation of tuber starches, there is a growing interest in non-traditional resources such as ulluco (*Ullucus tuberosus*) [103], oca (*Oxalis tuberosa*) [94], chufa (*Cyperus esculentus*) [172], and *Neorautanenia mitis* [89]. These tubers stand out for their key functional properties, such as their thickening, gelling, and stabilizing capacity, as well as their biological value as anti-inflammatory, antioxidant, and anticancer agents. From an economic viewpoint, the application and transformation of these tubers significantly contribute to the valorization of underutilized plant species, boosting the local economy of developing countries such as Bolivia, Chile, Ecuador, Peru, and Colombia, in addition to preserving traditional cultivation practices.

Regarding the contemporary legislative framework, starches are regulated globally under the Codex Alimentarius (Codex STAN 192–1995) [201]. Native starches are considered ingredients, whereas chemically modified ones are perceived as food additives; therefore, an INS identification must be included. This denomination constitutes a considerable limitation, as it generates a perception of artificiality or ultra-processing for the average consumer. The European Union presents a similar situation, where, in addition to strict nomenclature, the application of modified starches is only permitted in products like soups, sauces, and flavored yogurts, prohibiting their free application to products outside the norm. In the United States, the FDA’s limitations are less strict; however, the American consumer’s perception surrounding the denomination “Modified” clouds the panorama due to the association they make with the term “Genetically Modified (GM),” which represents a challenge for the development of the starch modification area and its application in microencapsulates.

It indicates that physical and enzymatic modifications have gained preference in the technological and industrial spheres. This trend responds to the growing inclination of researchers toward products with “green labels,” which opens possibilities for their application in commercial products for habitual consumption, such as breads, dairy products, and frozen foods, which require natural, sustainable, and nutritious ingredients and processes. These modification techniques not only guarantee the quality of modified starches and their applications in food matrices but also promote an environmentally responsible production chain [45].

## 6. Conclusions

In conclusion, this review consolidates the potential of modified tuber starches as versatile and sustainable biopolymers, whose technofunctionality is enhanced through the application of physical, chemical, or biochemical treatments, overcoming key technological limitations such as low thermal resistance and high retrogradation present in their native forms. The compiled evidence demonstrates that these modifications are crucial for their successful application as wall materials in the encapsulation of bioactive compounds, showing high efficiency in techniques such as spray drying, freeze-drying, electrospinning, and nanoemulsification. Nevertheless, the final performance of the encapsulate critically depends on the synergy among the botanical origin of the starch, the modification method, and the chemical compatibility with the active agent (core). Despite this promising profile, application on an industrial scale presents significant challenges related to process scalability, production costs, and the scarce development of legislation and the regulatory framework, where modified starches continue to be categorized as “additives” and often only consider those modified by chemical methods. Thus, this study provides a novel perspective to the scientific community by focusing specifically on the effects of tuber starch modification and its application in encapsulation, a niche that has been less explored compared with more general research, such as that on cereal and legume starches [202,203] and their nutritional potential, or that concerning the search for non-conventional sources, such as agro-industrial byproducts [5,48]. Therefore, the present work positions itself as a specific source of information whose future perspectives focus on adequately evaluating the properties of tuber starches, optimizing green label modification methods (physical and enzymatic), and valuing non-conventional sources. Consequently, this constantly evolving field invites the research community to address the study with a sequential and comprehensive approach, starting by evaluating the structural changes and internal phenomena generated by the different modifications in starches, along with the exhaustive evaluation of their techno-functional properties. Direct efforts toward the practical application of these biopolymers as protective agents for bioactive compounds are recommended, optimizing modification methods and integrating advanced engineering techniques that contribute to the development of a more sustainable, healthy, and innovative food science.

## Figures and Tables

**Table 2:** Parameters of physical modification and their effects on the properties of tuber starches.

Tuber	Type of Modification	Parameters	Effects on the Starch Characteristics	Reference
Amazonian white yam (*Dioscorea* sp.)	UV radiation	- Wavelength: 256 nm - t: 1 h.	Morphological alteration (irregular aggregates with increased porosity) ↓ Granule size (21.1 µm) ↓ Gelatinization enthalpy (3.2 J/g) ↑ Breakdown (1117 mPa·s) ↓ Viscosity (3109.9 mPa·s) ↓ Hysteresis area (89.34%)	[75]
	Microwave radiation	- Power: 700 W - t: 5 min	Morphological alteration (swollen and gelatinized granules) ↑ Particle size (36.0 µm) ↑ Viscosity (3816.1 mPa·s) ↓ Setback (2247 mPa·s) ↓ Relative crystallinity (11.8%)	
*Neorautanenia mitis* tuber	Pre gelatinization	Pregelatinized - Solvent: Water at 20% concentration (*w*/*v*) - T°: 55 °C - t: 15 min Drying - T°: 60 °C - t: 48 h.	Morphological alteration (rough surface). ↑Swelling capacity (maximum of 112.5% at 80 °C) ↑ Water absorption capacity (59.0%) ↑ Retrogradation (281.92 cP) ↓ Gelatinization enthalpy (5.27 J/g K) ↓ Peak viscosity (585.17 cP)	[89]
Palmyra tuber (*B. flabellifer* L.)	Pre gelatinization	Pregelatinized - Solvent: water at 1:1 (*w*/*v*) - T°: 63 °C - t: 5 min Drying - T°: 40 °C - t: 24 h.	Morphological alteration (irregular aggregates) ↓ Particle size (5.35 µm) ↓ Amylose content (9.2%) ↓ Viscosity (4.0 $mm^{2}$/s) ↓ Swelling (2.72 SP) ↑ Solubility (9.67%) ↑ Gelatinization temperature (82.27 °C)	[77]
Taro (*Colocasia esculenta*)	Heat–moisture treatment	Hydration: - Solvent: 25% (*w*/*w*) water - T°: Ambient - t: 48 h. Drying - T°: 120 °C - t: 3 h	↓ Free amylose ↑ Polymerization (DP > 100)	[45]
	Autoclave–cooling cycles	Hydration: - Solvent: water (1:2) Autoclaving - T°: 121 °C - t: 15 min Cooling: - T°: 4 °C - t: 24 h - Cycles: 2	Formation of type 3 resistant starch ↑ Retrogradation ↑ Heat resistance ↓ Polymerization (DP = 40–60)	

**Table 3:** Parameters and effect of chemical modifications on the characteristics of tuber starches.

Tuber	Modification Type	Parameters	Effects of Starch Characteristics	Ref.
Taro (*Colocasia esculenta*)	Esterification using OSA	Succinylation: - RC: 3% OSA (*w*/*w* starch) - pH: 8.5 (with 1 M NaOH) - RT: 6 h - Washing: Water and acetone	↑ Emulsifying capacity (DS = 0.022) ↑ Oxidative stability (PI = 6.20 meq-O_2_/kg) ↓ Digestibility (17.31% resistant starch)	[100]
Native Potato, “*Peruanita*” variety”	Esterification using OSA	Succinylation: - RC: 3% OSA (*w*/*w* starch) - pH: 8.5 (with 0.1 M NaOH) - RT: 6 h - Neutralization: pH 7.0 (with citric acid) - Washing: water - Centrifugation: 3000 rpm × 10 min. - Drying: 40 °C × 12 h.	↑ Colloidal stability (ζ-potential = −37.35 mV) ↓ Solubility (76.14–82.89%) ↑ Particle size (33.9 µm)	[101]
Potato (*Solanum tuberosum*)	Acid hydrolysis with modification	Hydrolysis: - RC: H_2_SO_4_ al 3 N (1:5 *w*/*v*) - RT: 3 h (at 60 °C) - Neutralization: Saturated Na_2_CO_3_ - Centrifugation: 2500 rpm × 5 min. - Washing: Water and ethanol - Drying: 70 °C × 12 h.	↓ Hydrolysis (DE = 2) ↓ Viscosity ↑ Thermal stability (160 °C) Gelatinization onset temperature: 120 °C × 10 min.	[55]
	Citric acid esterification	Esterification - RC: 30% citric acid (*w*/*w* starch) - pH: 3.5 (with 10 M NaOH) - RT: 14 h - Drying: 60 °C × 8 h. - Washing: Distilled water - Dry reaction: 130 °C × 5 h.	↑ Emulsion stability ↑ Solubility (for aqueous systems) Pseudoplastic behavior (n < 1)	[102]
Olluco (*Ollucus tuberosus*)	Esterification using OSA	Esterification - RC: 3% OSA (*w*/*w* starch) - pH: 8.5–9.0 (with 1 M NaOH) - RT: 6 h (25 °C) - Neutralization: 1 M HCl. - Drying: 60 °C × 8 h. - Washing: Distilled water and acetone.	↓ Peak gelatinization temperature (55.50 °C) ↓ Enthalpy (10.17 J/g) ↓ Pasting temperature (53.9 °C). ↑ Peak viscosity (3.65 Pa·s) Altered β-type X-ray diffraction pattern Functional properties (emulsion and stability)	[103]
Oca (*Oxalis tuberosa*)	Esterification using OSA	Esterification - RC: 3% OSA (*w*/*w* starch) - pH: 8.5–9.0 (with 1 M NaOH) - RT: 6 h (25 °C) - Neutralization: 1 M HCl. - Drying: 60 °C × 8 h. - Washing: Distilled water and acetone.	↓ Peak gelatinization temperature (54.6 °C) ↓ Enthalpy (8.60 J/g) ↓ Pasting temperature (54.9 °C). ↑ Peak viscosity (3.81 Pa·s). Altered β-type X-ray diffraction pattern	
Oca (*Oxalis tuberosa*)	Esterification with OSA + crosslinking with sodium trimetaphosphate	Esterification - RC: 3% OSA (*w*/*w* starch) - pH: 8.5 (with 1 M NaOH) - RT: 6 h (at 20 °C) - Neutralization: 1 M HCl. - Washing: Distilled water - Drying: 40 °C × 24 h. Crosslinking - RC: STMP (0.25–2.5% *w*/*w*) - pH adjustment: 10, 1 M NaOH - RT: 1 h (at 45 °C) - Neutralization: 1 M HCl (pH 5.5) - Washing: Distilled water	↓ Amylose content (19.61–23.18%) ↓ Transmittance (90.87–92.73%) ↓ Gelatinization enthalpy (3.48–1.95 J/g) ↑ Crystallinity (35.46–47.33%) ↑ Lipid absorption index (280.45–370.12%) ↑ Peak viscosity (1351.46–1558.62 mPa·s) ↑ Water absorption index (5.80–7.95 g/g) ↑ Water solubility index (3.50–6.40%) ↑ Swelling power (8.20–11.20 g/g)	[94]

**Note**: DE = dextrose equivalent, RC = reaction conditions, RT = reaction time, OSA = octadecyl succinic anhydride.

**Table 4:** Parameters and effects of biochemical modifications on the characteristics of tuber starches.

Tuber	Modification Type	Parameters	Effects of Starch Characteristics	Ref.
*Potato (Solanum tuberosum*)	Lactic Fermentation	- Bacterium: *L. plantarum* CGMCC 14177 - T°: 34 °C - RH: 80% - t: 24 h - Inoculum: 2% (*v*/*v*) of starter culture (10^8^ UFC/mL)	Surface with pits or dents ↓ Available Carbohydrates (21.48%) ↓ Enthalpy (6.26 J/g) ↓ Peak Viscosity (2882 cP). ↑ Amylose Content (34%) ↑ Setback Viscosity (1452 cP) ↑ Crystallinity (27.6%) ↑ Hardness (408.5 g) ↑ Chewiness (393.7 g)	[115]
Sweet potato (*Ipomoea batatas* L.)	Lactic acid fermentation	- Bacterium: *L. callosus*, *S. lactis*, and *C.* sp. - Yeast: *P. membranifaciens* - T°: 30 °C - t: 48 h - Inoculum. Mixed culture (cassava + coconut tuba + yogurt whey)	↓ Amylose content (24.1–27.6%) ↓ Average molecular weight (1.27–1.89 Mw × 10^7^ g/mol) ↓ Enthalpy (11.7–12.1 J/g) ↓ Final viscosity (1237–3185 cP) ↑ Gelatinization onset temperature (68.3–68.7 °C) ↑ Gel hardness (36–136 g) ↑ Gel cohesiveness (0.62–0.72)	[116]
Talas Bentul (*Xanthosoma sagittifolium*)	Enzymatic Fermentation	- Yeast: *Tape yeast* - T°: 27–30 °C - t: 12–48 h.	↑ Whiteness degree (up to 24 h: 87.5–90.3%) ↓Swelling Power (20.6–20.1 g/g) ↑ Viscosity (up to 24 h: 13.5–14.4 cP)	[114]
		- Yeast: *Saccharomyces cerevisiae* - T°: 27–30 °C - t: 12–48 h.	↑ Whiteness degree (84.2–87.1%) ↑ Amylose content (27.7–29.6%) ↑ Viscosity (28.2–29.6%) ↑Swelling Power (20.10–20.70 g/g)	
Potato (*Solanum tuberosum*)	Enzymatic Modification	- Enzyme: 4,6-α-glucanotransferase (StGtfB) - Buffer: sodium phosphate solution (pH 6.0) - T°: 40 °C - t: 2 h. - Drying: 60 °C × 8 h. - Enzyme concentration: 1–5 U/g of substrate	↑ Short-chain ratio–DP < 13 (77.11%) ↑ Polydispersity index (3.47) ↓ Molecular weight $(4.39\;\times \;10^{6}$ g/mol) ↓ Consistency index (0.06 $Pa.s^{n}$) ↓ Yield stress (0.14 Pa) ↓ RDS-Rapid digestion (0.14 Pa) ↑ SDS-Slow digestion (53.22%)	[117]
	Combined Physico-Enzymatic Modification	Physical Treatment - Freezing: −20 °C for 24 h. - Thawing: 25 °C for 2 h. - Cycles: 3 and 5 cycles Enzymatic Modification - Enzyme: Activated α-amylase - Activation: Ultrasound at 64.5 W, 25 + 40 kHz for 5 min - Buffer: Calcium chloride solution (pH 6.5) - T°: 90 °C	↑ Porosity (42.3%) ↑ Solubility (35.2%) ↓ Particle size (22.41 µm) ↓ Crystallinity (19.81 %) ↓ Peak viscosity (1650 cP) ↓ Retrogradation (10.8%) ↓ Swelling capacity (6.1 g/g)	[118]

**Table 5:** Application of the spray-drying encapsulation technique using tuber starch.

Tubers	Parameters	Effects on the Characteristics of the Microencapsulates	Ref.
Taro (*Colocasia esculenta*)	- WM: autoclave-cooling modified starch (2 cycles) - CM: *Lactobacillus plantarum* SU-LS 36 - $T{{}^{\circ}}_{in}$: 125 °C - $T{{}^{\circ}}_{out}$: 50 °C - FF: 4 mL/min - $\mathrm{AF}:\;20\;m^{3}/h$ - Pressure: 0.196 MPa	↑ Encapsulation efficiency (89.83%) ↑ High-temperature survival to 70 °C × 30 min (52.3%) ↓ Storage reduction rate (0.41 log CFU/g/week) ↓ Microencapsulation yield (40.19%) Spherical morphology (50–60 µm)	[45]
	- WM: OSA-succinylated starch - CM: Pomegranate seed oil - $T{{}^{\circ}}_{in}$: 170–190 °C - $T{{}^{\circ}}_{out}$: 99–117 °C - FF: 5 mL/min - Feed solid concentration: 15–25% - Oil/wall material ratio: 1:3 a 1:4	↓ Encapsulation efficiency (22.81–61.09%) ↓ Peroxide index (6.20 $meq-O_{2}$/Kg) ↓ Moisture content (1.26%) ↓ Bulk density (230 kg/$m^{3}$) ↓ Water activity ($a_{w}$ = 0.08) ↓ Water solubility (9.81%) ↓ Bioaccessibility–intestinal release (49.8%) ↓ Process yield (23.1%) Spherical morphology (10.16 ± 3.36 µm)	[100]
Potatoes (*Solanum tuberosum*)	- WM: OSA-succinylated starch + tara gum - CM: Propolis ethanolic extract - $T{{}^{\circ}}_{in}$: 120 °C - $\;T{{}^{\circ}}_{out}$: 65 °C - FF: 650 L/h. - Pump rate: 30% - Nozzle size: 0.7 mm.	↓ Encapsulation efficiency (24.66–56.74%) ↓ Process yield (55.0–57.03%) ↓ Water activity ($a_{w}$ = 0.26–0.34) ↓ Moisture content (5.26–7.03%) ↓ Particle size (36.7–53.7 µm) ↑ Solubility (76.14–82.89%) ↑ Antioxidant capacity (8.71–20.25 µmol ET/g) Sustained release of phenols between 7 and 13 h (8.13–12.36 mg GAE/g)	[101]
Papa (*Solanum tuberosum*)	- WM: Acid-hydrolyzed starch H_2_SO_4_ - CM: gallic acid (0.1–10.0 g/100 g) - $T{{}^{\circ}}_{in}$: 160 ± 5 °C - $T{{}^{\circ}}_{out}$: 75 ± 5 °C	↑ Encapsulation efficiency (65–79%) ↓ Porosity (1.2–4.9 $\times \;10^{3}\;cm^{2}$/g) ↓ Surface area (0.472–1.296 $m^{2}$/g) ↓ Water activity ($a_{w}$= 0.170–0.187) Porous spherical morphology (PSM)	[55]
	- WM: Citric acid-esterified starch - CM: Whey protein (lutein) - Core/wall ratio: 10:0, 9:1, 7:3, 5:5, 3:7, 1:9, and 0:10 - $T{{}^{\circ}}_{in}$: 185 ± 5 °C - $T{{}^{\circ}}_{out}$: 85 °C ± 5 °C - FF: 400 mL/h.	↓ Particle size (1–180 µm) ↓ Residual moisture (1.03–2.32%) ↓ Process yield (7.42–55.97%) ↑ Encapsulation efficiency (26.43–89.36%) ↑ Water solubility (49.71–77.44%) ↑ Lutein retention (56.23–91.54%)	[102]
Oca (*Oxalis tuberosa*)	- WM: OSA-modified starch in the pink oca - CM: phenolic extract of purple mashua - Emulsion ratio: 2–12% (*w*/*w*) - $T{{}^{\circ}}_{in}$: 120 a 160 °C - AF: 473 L/min - FF: 3 mL/min	↑ Encapsulation efficiency (35.29–84.31%) ↑ Total phenols content (1.96–7.84 mg/g) ↑ Antioxidant capacity (18.07–47.83 µmol Trolox/g) ↑ Solubility (4.34–9.12%) ↓ Water activity (0.24–0.44) ↓ Hygroscopicity (1.29–9.93%)	[46]
Olluco (*Ollucus tuberosus*)	- WM: OSA-modified starch - CM: Purple mashua phenolic extract (4% *w*/*w*) - $T{{}^{\circ}}_{in}$: 140 °C - AF: 473 L/min - FF: 3 mL/min	↑ Encapsulation efficiency (68.81 ± 1.05%) ↑ Total phenols content (4.57 ± 0.10 mg/g) ↑ Antioxidant capacity (36.03 ± 0.43 µmol Trolox/g)	

**Note**: WM = wall material; FF = feed flow; CM = core material; AF = air flow.

## Data Availability

No new data were created or analyzed in this study.
